# Swedish national survey on MR safety compared with CT: a false sense of security?

**DOI:** 10.1007/s00330-019-06465-5

**Published:** 2019-12-13

**Authors:** Boel Hansson, Johan Olsrud, Jonna Wilén, Titti Owman, Peter Höglund, Isabella M. Björkman-Burtscher

**Affiliations:** 1 Department of Medical Imaging and Physiology, Skåne University Hospital, Lund, Sweden; 2 Department of Diagnostic Radiology, Clinical Sciences, Lund University, Lund, Sweden; 3 Department of Radiation Sciences, Umeå University, Umeå, Sweden; 4 Department of Clinical Pharmacology, Clinical Sciences, Lund University, Lund, Sweden; 5 Department of Radiology, Clinical Sciences, Sahlgrenska Academy, University of Gothenburg, Gothenburg, Sweden

**Keywords:** Safety management, Magnetic resonance imaging, Risk assessment, Surveys and questionnaires, Patient safety

## Abstract

**Objectives:**

The objectives were to survey MR safety incidents in Sweden during a 12-month period, to assess severity scores, and to evaluate the confidence of MR personnel in incident-reporting mechanisms.

**Method:**

Data were collected within a web-based questionnaire on safety in clinical MR environments with CT for comparison. Data reported MR and CT safety incidents (human injury, material damage, and close calls), incident severity, and confidence of participants in incident-reporting systems.

**Results:**

The study population consisted of 529 eligible participants. Participants reported 200 MR and 156 CT safety incidents. Among MR incidents, 16% were given the highest potential severity score. More MR workers (73%) than CT workers (50%) were confident in being aware of any incident occurring at their workplace. However, 69% MR workers (83% for CT) were not aware of reported incidents at their hospitals.

**Conclusion:**

Safety incidents resulting in human injury, material damage, and close calls in clinical MR environments do occur. According to national risk assessment recommendations, risk level is high. Results indicated that MR personnel tend to a false sense of security, as a high proportion of staff members were sure that they would have been aware of any incident occurring in their own department, while in reality, incidents did occur without their knowledge. We conclude that false sense of security exists for MR.

**Key Points:**

*• Safety incidents in clinical MR environments still result in human injury and material damage.*

*• Severity level of MR incidents—assessed using Swedish national risk assessment recommendations—is high.*

*• Confidence of MR personnel in incident-reporting mechanisms is high, but reflects a false sense of security, as a high proportion of staff is unaware of reported incidents in the same workplace.*

## Introduction

For MR safety, three types of electromagnetic field exposure must be considered: the static magnetic field, the gradient magnetic field, and the radio-frequency field [1]. In some individuals, when moving through it, the static magnetic field causes short-term effects such as vertigo and nausea, but no serious adverse effects have been reported [2–4]. The hazard of projectiles, attracted by the static magnetic field, is the most dangerous risk in MR [5, 6]. Translational forces on ferromagnetic objects in and outside the body are more pronounced with today’s actively shielded magnets since the spatial gradient―the rate of change of the static magnetic field with respect to distance―is steep [7].

The time-varying gradient magnetic field present during image acquisition may lead to peripheral nerve stimulation, cause acoustic noise, and/or affect implants. When MR safety standards are adhered to, peripheral nerve stimulation can be kept below the level of concern [8], and injury due to acoustic noise [6, 9] or harmful induction of electrical currents in implants, such as pacemakers, can be prevented [10–13].

The radio-frequency coil transfers energy into the body and can cause heating. Currents are induced in electrically conductive tissue or implants, and heating may occur due to resistance to the current [7]. MR safety procedures are aimed at prevention of potential thermal risks in electrically conductive materials, at ensuring that tissues do not form electrically conductive loops, and at raising awareness of other factors (e.g., tattoos) that constitute a possible heating risk [14].

Safety-related incidents may increase as the number of scanners installed increases [15]. Although already in 1994 Boutin et al [16] had pointed out the importance of screening procedures, it was not until an MR-unsafe oxygen tank had killed a 6-year-old boy [17] that the first guidelines for MR safety were developed [14]. This tragic event was even preceded by two other projectile-related close calls, neither adequately communicated among local staff nor leading to re-evaluation of safety procedures [18].

Incident-reporting systems are essential to be aware of incidents, understand their causes, and prompt action for prevention avoiding future human suffering and also saving hospital costs [19–22].

MR in clinical practice is still not completely safe. Thus, the aims of this national web-based survey in Sweden were to survey MR safety incidents that occurred over a 12-month period, to assess incident severity and to evaluate confidence of MR personnel in incident-reporting mechanisms. Further, we compared with CT personnel as a control group.

## Material and methods

### Survey

This study was part of a national web-based questionnaire focusing on the working environment for MR workers and on safety issues for patients and personnel with CT for comparison.

MR vendors provided a list of installed bases in Sweden and personal contact was made with each site to identify a person responsible for MR and/or CT who could distribute information about the study and post a link to the questionnaire. Data were collected over a 6-month period, and personnel scanning to any degree with MR and/or CT were eligible to participate; thus, the survey targeted primarily MR and CT radiographers.

### Background population

Sweden, a country with approximately 10 million inhabitants, had at the time of the survey 92 MR sites for human imaging (information collected from vendors and verified by personal contact) with more than 225 scanners (information collected from survey). The number of MR users, defined as personnel regularly scanning humans and thus mainly referring to MR radiographers, was for Sweden estimated to be approximately 620 (information collected from contact persons at all MR sites). The equivalent estimate of CT users, at the MR sites that also had CT, was approximately 1300.

### Study population and workplaces

The following demographic data were collected: numbers of MR and/or CT workers who responded; age; gender; full-time or part-time work; percentage of full time (full time = 40 h/week) dedicated to work with MR, CT, and other modalities (e.g., ultrasound, conventional radiology) or administration; modality experience; number and type(s) of hospitals represented and their installed base; and patient demographics (e.g., clinical, research, level of care, care burden).

### Safety data

Data on safety incidents focused on human injuries, material damage, and close calls. The participants stated whether they were aware of any safety-related incidents that had occurred at their hospital during the last 12-month period before participation in the survey, including a voluntary free-text description of the incidents, to classify incidents and exclude double reports. Free-text comments were not mandatory to allow protection of the integrity of the participants and to decrease the risk of under-reporting due to fear of recognition, an issue emphasized during ethical evaluation of the study design. Participants were also asked if they were confident that any safety incidents that might have occurred at their workplace would have come to their attention (confidence in incident-reporting mechanisms). Questions on safety were repeated for MR and CT for comparison, allowing participants working with both modalities to fill in a complete set of safety questions for both modalities. There is no national register for safety incidents, and hospitals are only encouraged to report any preventable serious incidents that have or might have led to human injury to the health and social care inspectorate, a government agency.

### Data evaluation and statistics

Demographic data on the study population and on safety incidents are reported with descriptive statistics. Safety incidents resulting in human injury were first processed with an assessment of a *severity score* based on free-text comments and adapted national recommendations for risk assessment and incidence analysis [23]. The score is based on the National Patient Safety Improvement Handbook [24], and only considers human injury. Mainly, immediate consequences were expected to be mentioned in the free-text comments, as personnel at radiology departments usually has not the opportunity to follow up on long-term outcome after incidents, which might differ for very severe incidents, where feedback loops are expected to be more efficient, not the least due to possible legal consequences. Further, all safety incidents were scored with a *potential severity score* defining the potential worst-case scenario outcome of a similar incident. Scores were as follows: 1 = minor (discomfort or insignificant injury); 2 = intermediate (transient sensory, motor, physiological, intellectual, or mental disability; extended care episode; or increased care level); 3 = significant (persistent moderate sensory, motor, physiological, intellectual, or mental impairment; extended care episode; or increased care level); and 4 = catastrophic outcome (death, persistent major sensory, motor, physiological, intellectual or mental disability). Scoring was performed during a consensus discussion regarding each safety incident by the head MR safety physicist (JO), the head MR safety research radiographer (TO), the responsible research radiographer (BH), and the Principal Investigator of the study, a neuroradiologist with 20 years of experience (IBB).

McNemar’s chi-square test with continuity correction was used to evaluate potential differences in confidence in incident-reporting mechanisms regarding MR and CT in individuals working with both modalities. Mann-Whitney *U* test and Wilcoxon signed-rank test were used for evaluation of group differences.

## Results

### Study population and workplaces

**Fig. 1:**
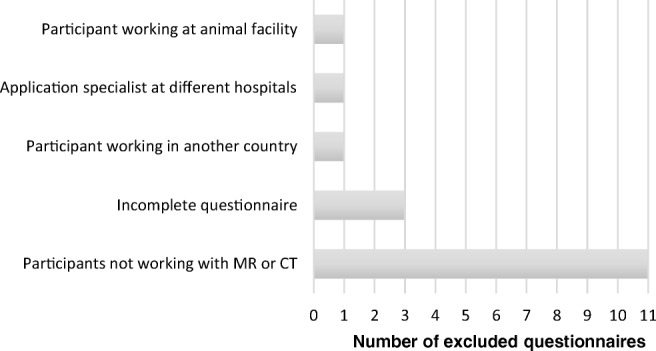
Reasons for exclusion of 17 questionnaires (total submitted 546)

In total, 529 eligible answers were registered after exclusion of 17 questionnaires submitted incompletely or by participants who were not eligible. Figure 1 gives numbers and reasons for exclusion of submitted questionnaires. Of the 529 participants, 415 (78%) were women (mean age 46 years, median 48, range 23–66), 112 (21%) were men (mean age 43 years, median 43, range 25–65), and two participants did not define their gender. Only 7 participants were not radiographers (5 biomedical analysts, 1 physicist, and 1 nurse).

**Fig. 2:**
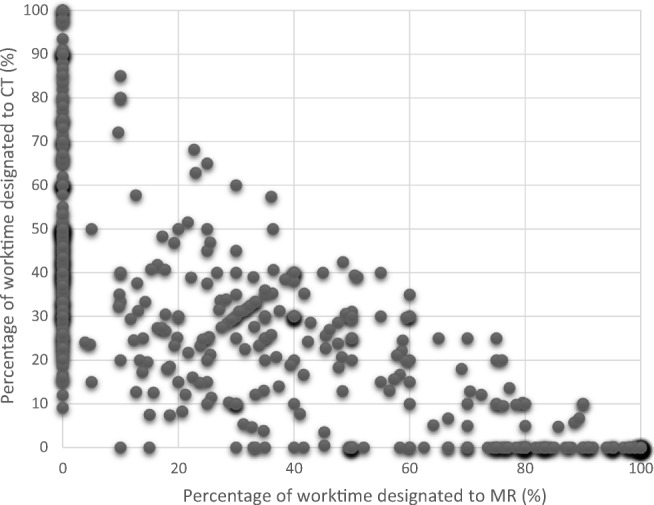
Self-estimated percentage of total work time designated to MR and/or CT for the 529 participants

Of the participants, 345 worked part time or full time with MR, 392 worked part time or full time with CT, 137 with MR but not CT, 184 with CT but not MR, and 208 with both MR and CT. Part-time work with other modalities is not reported here due to its limited relevance. When the work time was compared, MR had a significantly higher level of work hours than CT (Wilcoxon signed-rank test, *p* = 0.007). Table 1 is a summary of data on self-estimated percentage of total work time that was designated by the 529 participants to the two modalities under investigation, MR and CT, and Fig. 2 shows the individual distribution for the participants.

**Table 1:** Self-estimated percentage of total work time designated by participants to MR or CT

	Participants (*n*) working with modality				Estimated percentage of the full-time equivalent of 40 h/week designated to modality (mean %; range)
	0–100%	< 50%	50–90%	> 90%	
	Of the full-time equivalent of 40 h/week				
MR	345	165	113	67	56; 4–100
CT	392	295	86	11	39; 4–100
MR but not CT	137	9	61	67	83; 10–100
CT but not MR	184	102	71	11	51; 9–100

The normal working hours in Sweden for radiographers are 40 h per week, but individual working hours may vary due, for example, to part-time contracts, or part-time sick leave or parental leave. Fifteen participants reported that they worked < 75% of normal working hours, 80 participants worked 75–90% of normal working hours, and 434 participants worked 91–100% of normal working hours (40 h/week).

Mean experience for participants working with MR was 8 years (median 14; range 0–32 years) and mean CT experience was 12 years (median 10; range 0–38 years).

Prior to the survey, we established by personal contact the number of hospitals with MR units to be 92 in Sweden. The 529 participants in the survey worked at 86 hospitals (9 private); 167 (32%) of them worked at 12 hospitals affiliated to the seven medical universities in the country. The estimated response rate of MR workers was approximately 60%. The survey covered most MR scanners in the country, as all large hospitals were covered and the majority non-covered by the survey (*n* = 11) were small private MR facilities (*n* = 7). The participants working with MR in the study therefore worked at 81 hospitals, entailing approximately 225 MR scanners; and the participants working with CT worked at 84 hospitals with 253 CT installations.

The numbers of MR and CT scanners at each hospital or healthcare unit varied between 1 and 7 for MR (median 2) and 1–6 for CT (median 3). The most common MR scanner was a 1.5-T scanner (334 users), followed by 3-T installations (133 users). The most common CT scanner was 64-slice scanner (288 users), followed by 128-slice scanner (122 users).

**Fig. 3:**
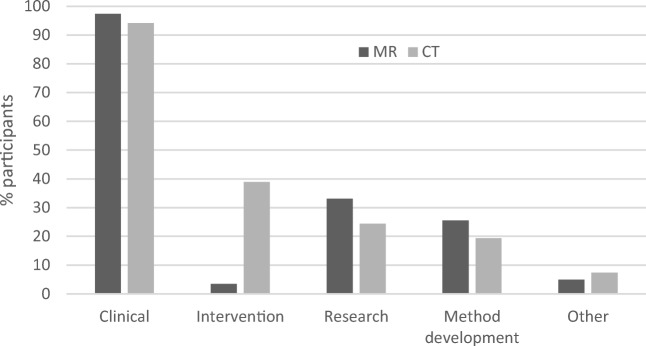
Percent of participants working with clinical examinations, research, interventions, method development, and/or other unspecified tasks regarding MR and CT

**Fig. 4:**
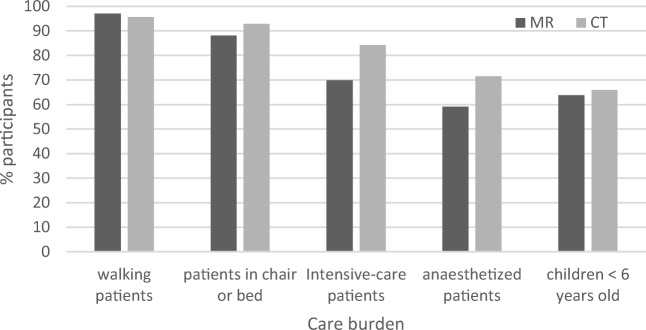
Percent of participants working with the different care burden categories in MR and CT

Figure 3 shows the percentage of participants working with clinical examinations, research, interventions, method development, and other unspecified tasks regarding the modalities in question. MR and CT showed similar distributions except for intervention, which was much more common in CT than in MR. For MR, the estimated percentage of patients with higher care burden was slightly lower than that for CT (Fig. 4).

### Safety incidents

Altogether, 200 MR safety incidents and 156 CT safety incidents were reported by the 529 participants. The numbers of human injuries, material damage, and close calls related to MR and CT are detailed in Table 2, together with information on multiple reporting of specified incidents, the number of participants reporting the incidents, the number of hospitals affected, and the number of participants working with the modality in question at these hospitals. Exclusion of multiple reporting was performed based on evaluation of the free-text comment, and reduced the number of specified incidents by a mean of 33% for MR and 19% for CT (Table 2).

**Table 2:** Safety incidents reported by 345 MR workers at 81 hospitals and 392 CT workers at 84 hospitals

Safety incident and modality evaluated		Reported incidents (*n*_tot_; *n*_spec_; *n*_spec_ex_)	Hospitals (*n*) with reported incidents	Participants not reporting incidents, *n* (%)	Participants *n* (%) working at hospitals			
					With reported incidents			Without reported incidents
					Total	Reporting an incident	Not reporting incidents	
HI	MR	21; 18; 11	11	326 (94)	87	19 (22)	68 (78)	258
	CT	64; 30; 25	23	359 (92)	177	33 (19)	144 (81)	215
MD	MR	50; 34; 21	15	308 (89)	116	37 (32)	79 (68)	229
	CT	39; 18; 14	15	369 (94)	152	23 (15)	129 (85)	240
CC	MR	129; 93; 65	33	263 (76)	201	82 (41)	119 (59)	144
	CT	53; 15; 12	17	369 (94)	145	23 (16)	122 (84)	247
Total	MR	200; 145; 97	37	306 (89)	220	39 (18)	181 (82)	125
	CT	156; 63; 51	34	329 (84)	248	63 (25)	185 (75)	144

*HI*, human injury; *MD*, material damage; *CCs*, close calls; *n*_tot_, *total* number of reported incidents; *n*_spec_, number of incidents further *specified* with free-text comment in questionnaire; *n*_spec_ex_, number of *specified* incidents *excluding* multiple reporting (further detailed in Table 3), as identified by hospital affected and description of incident

Details of specified reported safety incidents are given in Table 3, together with severity scores of the incidents. The pattern of incidents that were further specified by participants and evaluated after exclusion of multiple reporting differed between MR (*n* = 97) and CT (*n* = 51). MR users focused mainly on incidents related to the static magnetic field, the radio-frequency field, and the gradient magnetic field (projectiles, implants, and burns) (*n* = 92), whereas CT users focused on radiation issues (*n* = 3) and complications related to application of contrast media (*n* = 12), a topic that was not at all touched upon by any of the MR users. Incidents related to ergonomics (*n* = 41), with a mixture of heavy lifts of equipment or patients, and clamping and squeezing incidents involving equipment, affecting patients and personnel were reported for both modalities but more often by CT users (*n* = 36) than by MR users (*n* = 5). Material damage and close calls were more often reported by MR users than by CT users (Table 2); however, human injuries were more common for CT. Although not specifically requested, participants who used the voluntary free-text option commented about whom they regarded to be responsible for MR safety incidents: personnel from departments other than radiology (44 incidents), personnel from radiology (13 incidents), or a patient or relative (16 incidents); but for 24 incidents, the responsible party was unspecified.

**Table 3:** Numbers of safety incidents (*n*), which were further specified by participants in voluntary free-text comments, grouped according to cause and evaluated regarding severity score (SS) for actual human injuries and potential severity score (PSS) based on worst-case scenarios for all safety incidents. Scores range from 1 (minor) to 4 (catastrophic)

	MR							CT						
	Human injury			Material damage		Close call		Human injury			Material damage		Close call	
	*n*	SS	PSS	*n*	PSS	*n*	PSS	*n*	SS	PSS	*n*	PSS	*n*	PSS
Burns (total)	*5*^a^	*2*	*3*	*0*	*–*	*0*	*–*	*0*	*–*	*–*	*0*	*–*	*0*	*–*
Projectile (total)	*3*	*1–2*	*3*	*19*	*2–4*	*57*	*2–4*	*0*	*–*	*–*	*0*	*–*	*0*	*–*
Small, blunt	0			3^b^	2	15^c^	2							
Small sharp/median size	3^d^	1–2	3	15^e^	3	31^f^	3							
Large/heavy metal	0			1^g^	4	11^h^	4							
Implant (total)	*0*	*–*	*–*	*0*	*–*	*8*	*2–4*	*0*	*–*	*–*	*0*	*–*	*0*	**–**
Pacemaker						3	4							
Splinter close to eye						1	3							
Other						4^i^	2							
Ergonomics (total)	*3*^*j*^	*1*	*2*	*2*^*k*^	*2*	*0*	*–*	*14*^l^	*1–2*	*1–2*	*14*^m^	*2*	*8*^n^	*2*
Contrast medium (total)	*0*	*–*	*–*	*0*	*–*	*0*	*–*	*9*	*1–4*	*1–4*	*0*	*–*	*3*	*1–4*
Air injection													2	4
Extravasation								6	1	1			1	1
Needle-stick								2	1	1				
Adverse reaction								1^**o**^	4	4				
Radiation dose								*2*^p^	*1*	*1*	*0*	*–*	*1*	*1*
Total *n*/max. score	*11*	*2*	*3*	*21*	*4*	*65*	*4*	*25*	*4*	*4*	*14*	*2*	*12*	*4*

Severity scores: 1 = minor (discomfort or insignificant injury), 2 = intermediate (transient sensory, motor, physiological, intellectual, or mental disability; extended care episode; or increased care level), 3 = significant (persistent moderate sensory, motor, physiological, intellectual, or mental impairment; extended care episode; or increased care level), 4 = catastrophic outcome (death or persistent major sensory, motor, physiological, intellectual, or mental disability). Italics indicate total

Short explanations of objects/actions involved, with number of incidents given in parenthesis (*n*): *a*, skin-skin contact or loop (1), skin-coil contact (3), unspecified (1); *b*, glasses (1), hair clip (1), equipment part (1); *c*, hair pin (1), screw (1), keys (3), basket lid (1), phone (3), unspecified metal object in pocket (6); *d*, unspecified sharp object (1), unspecified magnetic object (1); wheelchair (1); *e*, scissors or knife (2), crutches (1), wheelchair (3), walker (2), ventilator/monitor (3), infusion pump (2), vacuum cleaner (1), cart (1); *f*, scissors or knife (7), crutches (2), laryngoscope (2), forceps (2), wheelchair (4), rescue stretcher (1), walker (6), ventilator/monitor (1), infusion pump (4), cleaning cart (1), cart (1); *g*, oxygen tank (1); *h*, oxygen tank (5), bed (6); *i*, leg prosthesis (1), tracheal tube (1), undefined metal implant (2); *j*, heavy lift/bumping into equipment (3); *k*, squeeze from equipment during table movement (2); *l*, strain injury due to heavy lift or bumping into equipment affecting personnel (10), squeeze of body part of personnel or patient from equipment (4); *m*, collision between patient bed and CT table, CT table lowered onto equipment―often patient bed, clothes, or equipment stuck during table movement (14); *n*, clothes or equipment close to getting stuck/squeezed during table movement in any direction (2), body parts close to getting squeezed during table movement or patient transfer (2), close to bumping into equipment (2), risk of strain injury due to heavy lift (1), risk of patient falling when leaving the table before it was lowered as intended (1); *o*, anaphylactic reaction to contrast medium with fatal outcome (1); *p*, radiation dose increased due to re-scan (2)

**Fig. 5:**
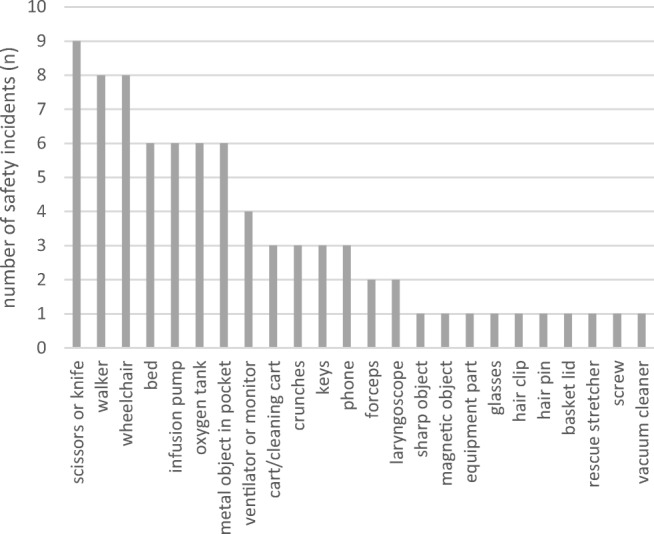
Short descriptions of projectiles involved in safety incidents (*n*) specified by participants in voluntary free-text comments

Assessment of severity of human injuries based on free-text descriptions gave severity scores of 1 to 3 for MR and 1 to 4 for CT, where the case with score 4 in CT refers to an anaphylactic reaction to contrast media with fatal outcome (Table 3). In all MR cases, potential―worst-case scenario―severity scores were higher than actual severity scores for human injuries, but unchanged for CT (Table 3). A summary of projectiles involved in safety incidents is given in Fig. 5.

### Confidence in incident-reporting mechanisms

Data on confidence in incident-reporting mechanisms are given in Table 4 for MR and CT workers. More MR workers than CT workers were confident that any safety incidents or close calls that might have occurred at their workplace would have come to their notice (mean for human injuries, material damage, and close calls: 73% of MR workers vs. 50% of CT workers). The proportion of MR workers who reported that they were not aware of any incidents at their workplace(s)―although other participants at their workplace had reported a safety incident―was somewhat lower (mean for human injuries, material damage, and close calls; 69%) than for CT workers (83%), but was very high in both groups despite the fact that both MR workers and CT workers had a high confidence in incident-reporting mechanisms.

**Table 4:** Numbers of participants (*n*; %) working with MR and/or CT who were confident (C) (bold) or were not confident (NC) (italics) that safety incidents that might have occurred at their own hospital, and involving their modality, would have come to their notice. Participants have been grouped according to whether or not they worked at hospitals with reported safety incidents and whether or not they reported incidents themselves

Participants working at hospitals with (yes) or without (no) reported safety incidents		Participants who did (yes) or did not (no) report any safety incidents	Participants (*n*) who were confident or not confident that safety incidents that might have occurred at their hospital would have come to their notice			
				Human injury	Material damage	Close call
MR	Yes	Yes	Confident	**15**	**32**	**63**
			Not confident	*4*	*5*	*19*
	Yes	No	Confident	**44**	**49**	**68**
			Not confident	*24*	*30*	*51*
	No	No	Confident	**208**	**181**	**101**
			Not confident	*50*	*48*	*43*
CT	Yes	Yes	Confident	**22**	**13**	–^a^
			Not confident	*11*	*10*	–^a^
	Yes	No	Confident	**62**	**47**	**49**
			Not confident	*82*	*82*	*73*
	No	No	Confident	**121**	**102**	**112**
			Not confident	*95*	*138*	*136*

^a^Due to a design problem in the questionnaire 14, participants, who reported a close call for CT, could not answer the question on confidence in incident-reporting mechanisms, and are thus not included in this evaluation

When we only considered participants working with both modalities (*n* = 208), the participants were significantly more confident that any safety incident that might have occurred at their workplace concerning MR would have come to their attention than regarding any safety incidents concerning CT (McNemar’s chi-square test with continuity correction; *p* < 0.0005 for human injury, material damage, and close call) (Table 5).

**Table 5:** Breakdown of participants working both with MR and CT (*n* = 208) who were confident (yes) or not confident (no) that any safety incidents that might have occurred at their workplace, concerning MR and CT, would have come to their attention

		Human injury		Material damage		Close call^a^	
Confident		MR					
		No	Yes	No	Yes	No	Yes
CT	No	50	39	49	43	70	38
	Yes	8	111	11	105	12	74

^a^Due to a design problem in the questionnaire 14 participants, who reported a close call, could not answer the question on confidence in incident-reporting mechanisms, and thus not included in this evaluation

## Discussion

As there is no national register for MR-related safety incidents in Sweden, this national survey was performed to gain a baseline overview of MR safety incidents that occurred in the country over a 12-month period. With a response rate of approximately 60% of all MR workers in Sweden, covering 90% of hospitals/facilities with MR units in Sweden, the 21 human injuries, 50 cases of material damage, and 129 close calls reported should cover the majority of MR safety incidents that occurred during the study period. At hospitals with incidents reported, 82% of MR users did not report having knowledge of any incident. An average of 61% of these users do not acknowledge the possibility that incidents might have occurred without their knowledge (65% for human injury, 62% for material damage, and 57% for close calls). This finding suggests that the healthcare system lacks functioning local incident-report systems assuring feedback to employees. This is also reflected by the rather low percentage of multiple reporting of specific incidents. Further, radiographers working with both MR and CT had significantly higher confidence in safety feedback regarding MR than CT incidents (*p* < 0.0005), illustrating that the efficacy of feedback mechanisms at a department may differ for modalities or be perceived differently. Reported MR incidents were mainly related to burns and projectiles, and were thereby related to the static magnetic field, or the radio-frequency field. Safety incidents related to ergonomic risks affected both patients and personnel and were mainly raised by CT users, which might be related to the larger throughput of patients at CT compared to MR. Reported close calls were predominant among MR safety incidents in this study, and we interpret this finding as reflecting a considerable awareness of safety risks and that safety practices and routines are in place as a necessary base for MR accident prevention. No lethal cases were reported for MR in this study. However, as a high number of close calls has been reported involving large items such as wheelchairs, ventilators, oxygen tanks, and beds, several lethal cases could have happened, inciting again on the necessity to adopt international recommendations for safety [13, 25–28]. This is also reflected in the fact that 15 (16%) MR safety incidents specified were given the highest potential—worst-case scenario—severity score. James reported that serious harm seems to be 10–20-fold more common than lethal harm [29].

MR-related risks are manifold, and incident prevention is complex and relies heavily on employees and thus patients become vulnerable in MR environments. Factors to minimize this vulnerability are continuously reviewed safety routines, staff education, feedback mechanisms on incidents, and the much more numerous close calls, and a change of culture towards learning from mistakes [11, 14, 16, 30–32]. Further, confidence in internal communication or local reporting systems might be much greater than the true usefulness of such routines, as shown in this study, and they need to be designed carefully [19]. Even if most MR sites in Sweden have safety screening checklists, advocated as single most effective measure for prevention [33], MR safety incidents still occur. Radiographers, radiologists, personnel from other departments accompanying patients to MR units, administrative staff, janitors, and firemen are important pieces of the MR safety puzzle, and leaving out one piece might jeopardize security and possibly lead to a catastrophe [28].

Based on our results showing that severe adverse events still exist, are poorly shared within the team, and are preventable, the following action steps are mandatory: (1) identify potential risk zones; (2) design specific educational programs dedicated to every category of professionals that work in or might visit MR sites with a focus on vigilance of MR personnel on potential mistakes made by other professionals, potential misunderstandings, or knowledge gaps; (3) state clear MR safety procedures including screening forms that are confirmed with an interview just before entering the MR-scanner room; and (4) facilitate rigorous but easily manageable incident-reporting systems with focus on prevention and learning from mistakes.

This nationwide survey well represents the MR environment in the country and is also internationally generalizable regarding many aspects. However, a survey of this kind can never claim to have complete coverage, and always leaves room for selection bias. Both underestimation and duplicate recording may have occurred. Incidents involving large and more hazardous objects might be more frequently reported than incidents with smaller and less hazardous objects. Also Mansouri et al [34] have pointed out that incident-reporting systems inherently suffer from under-reporting.

From an ethical point of view and to protect the integrity of employees, answers were completely anonymous and data are presented avoiding identification of specific facilities and their association with particular incidents. This limited the possibility of double-checking incidents, although similar descriptions of incidents in the free-text comments, concerning incidents occurring at the same hospital, have been detected and lead to exclusion of multiple reporting. However, to encourage participants not to hold back due to fear of individual identification, the free-text comment for specification of incidents was optional.

Our assessment of severity scores based on adapted national recommendations [23] can of course not be compared to a full-scale risk assessment based on detailed data concerning a specific incident. However, putting the reported and specified incidents into a severity context is of great importance, to highlight possible future risks and in MR safety-prevention work.

We chose to use CT for comparison in this study, in order to be able to put the results in a similar context, and due to the many similarities between the two work environments. Limitations of this approach are, for example, the significantly higher proportion of full-time working hours dedicated to the modality of MR, compared to CT, observed in this study and the known difference in throughput of examinations per hour for the two modalities.

In conclusion, this national survey has shown that safety incidents resulting in human injury, material damage, and close calls in clinical MR environments do occur. Risk level of these incidents is high. Results indicated that MR personnel tend to have a false sense of security, as a high proportion of staff members were sure that they would have been aware of any incident occurring in their own department, while in reality, incidents did occur without their knowledge. Using CT for comparison highlighted that individuals might consider safety and feedback differently, depending on the modality.
